# The sixth sense: how much does interictal intracranial EEG add to determining the focality of epileptic networks?

**DOI:** 10.1093/braincomms/fcae320

**Published:** 2024-09-27

**Authors:** Ryan S Gallagher, Nishant Sinha, Akash R Pattnaik, William K S Ojemann, Alfredo Lucas, Joshua J LaRocque, John M Bernabei, Adam S Greenblatt, Elizabeth M Sweeney, Iahn Cajigas, H Isaac Chen, Kathryn A Davis, Erin C Conrad, Brian Litt

**Affiliations:** Center for Neuroengineering and Therapeutics, University of Pennsylvania, Philadelphia, PA 19104, USA; Perelman School of Medicine, University of Pennsylvania, Philadelphia, PA 19104, USA; Center for Neuroengineering and Therapeutics, University of Pennsylvania, Philadelphia, PA 19104, USA; Department of Bioengineering, University of Pennsylvania, Philadelphia, PA 19104, USA; Department of Neurology, University of Pennsylvania, Philadelphia, PA 19104, USA; Center for Neuroengineering and Therapeutics, University of Pennsylvania, Philadelphia, PA 19104, USA; Department of Bioengineering, University of Pennsylvania, Philadelphia, PA 19104, USA; Center for Neuroengineering and Therapeutics, University of Pennsylvania, Philadelphia, PA 19104, USA; Department of Bioengineering, University of Pennsylvania, Philadelphia, PA 19104, USA; Center for Neuroengineering and Therapeutics, University of Pennsylvania, Philadelphia, PA 19104, USA; Perelman School of Medicine, University of Pennsylvania, Philadelphia, PA 19104, USA; Department of Bioengineering, University of Pennsylvania, Philadelphia, PA 19104, USA; Center for Neuroengineering and Therapeutics, University of Pennsylvania, Philadelphia, PA 19104, USA; Department of Bioengineering, University of Pennsylvania, Philadelphia, PA 19104, USA; Department of Neurology, University of Pennsylvania, Philadelphia, PA 19104, USA; Center for Neuroengineering and Therapeutics, University of Pennsylvania, Philadelphia, PA 19104, USA; Perelman School of Medicine, University of Pennsylvania, Philadelphia, PA 19104, USA; Department of Bioengineering, University of Pennsylvania, Philadelphia, PA 19104, USA; Department of Neurology, University of Pennsylvania, Philadelphia, PA 19104, USA; Department of Biostatistics, Epidemiology and Informatics, University of Pennsylvania, Philadelphia, PA 19104, USA; Department of Neurosurgery, University of Pennsylvania, Philadelphia, PA 19104, USA; Department of Neurosurgery, University of Pennsylvania, Philadelphia, PA 19104, USA; Corporal Michael J. Crescenz Veterans Affairs Medical Center, Philadelphia, PA 19104, USA; Center for Neuroengineering and Therapeutics, University of Pennsylvania, Philadelphia, PA 19104, USA; Department of Neurology, University of Pennsylvania, Philadelphia, PA 19104, USA; Center for Neuroengineering and Therapeutics, University of Pennsylvania, Philadelphia, PA 19104, USA; Department of Neurology, University of Pennsylvania, Philadelphia, PA 19104, USA; Center for Neuroengineering and Therapeutics, University of Pennsylvania, Philadelphia, PA 19104, USA; Department of Neurology, University of Pennsylvania, Philadelphia, PA 19104, USA

**Keywords:** intracranial EEG, spatial coverage, normative atlas, seizure onset zone, epilepsy surgery

## Abstract

Intracranial EEG is used for two main purposes: to determine (i) if epileptic networks are amenable to focal treatment and (ii) where to intervene. Currently, these questions are answered qualitatively and differently across centres. There is a need to quantify the focality of epileptic networks systematically, which may guide surgical decision-making, enable large-scale data analysis and facilitate multi-centre prospective clinical trials. We analysed interictal data from 101 patients with drug-resistant epilepsy who underwent pre-surgical evaluation with intracranial EEG at a single centre. We chose interictal data because of its potential to reduce the morbidity and cost associated with ictal recording. Sixty-five patients had unifocal seizure onset on intracranial EEG, and 36 were non-focal or multi-focal. We quantified the spatial dispersion of implanted electrodes and interictal intracranial EEG abnormalities for each patient. We compared these measures against the ‘5 Sense Score,’ a pre-implant prediction of the likelihood of focal seizure onset, assessed the ability to predict unifocal seizure onset by combining these metrics and evaluated how predicted focality relates to subsequent treatment and outcomes. The spatial dispersion of intracranial EEG electrodes predicted network focality with similar performance to the 5-SENSE score [area under the receiver operating characteristic curve = 0.68 (95% confidence interval 0.57, 0.78)], indicating that electrode placement accurately reflected pre-implant information. A cross-validated model combining the 5-SENSE score and the spatial dispersion of interictal intracranial EEG abnormalities significantly improved this prediction [area under the receiver operating characteristic curve = 0.79 (95% confidence interval 0.70, 0.88); *P* < 0.05]. Predictions from this combined model differed between surgical- from device-treated patients with an area under the receiver operating characteristic curve of 0.81 (95% confidence interval 0.68, 0.85) and between patients with good and poor post-surgical outcome at 2 years with an area under the receiver operating characteristic curve of 0.70 (95% confidence interval 0.56, 0.85). Spatial measures of interictal intracranial EEG abnormality significantly improved upon pre-implant predictions of network focality by area under the receiver operating characteristic curve and increased sensitivity in a single-centre study. Quantified focality predictions related to ultimate treatment strategy and surgical outcomes. While the 5-SENSE score weighed for specificity in their multi-centre validation to prevent unnecessary implantation, sensitivity improvement found in our single-centre study by including intracranial EEG may aid the decision on whom to perform the focal intervention. We present this study as an important step in building standardized, quantitative tools to guide epilepsy surgery.

## Teaser

Gallagher *et al.* report that spatial information about background intracranial EEG abnormalities improves upon non-invasive data collected before implantation to predict which patients will have focal onset seizures.

## Graphical Abstract

**Graphical Abstract:**
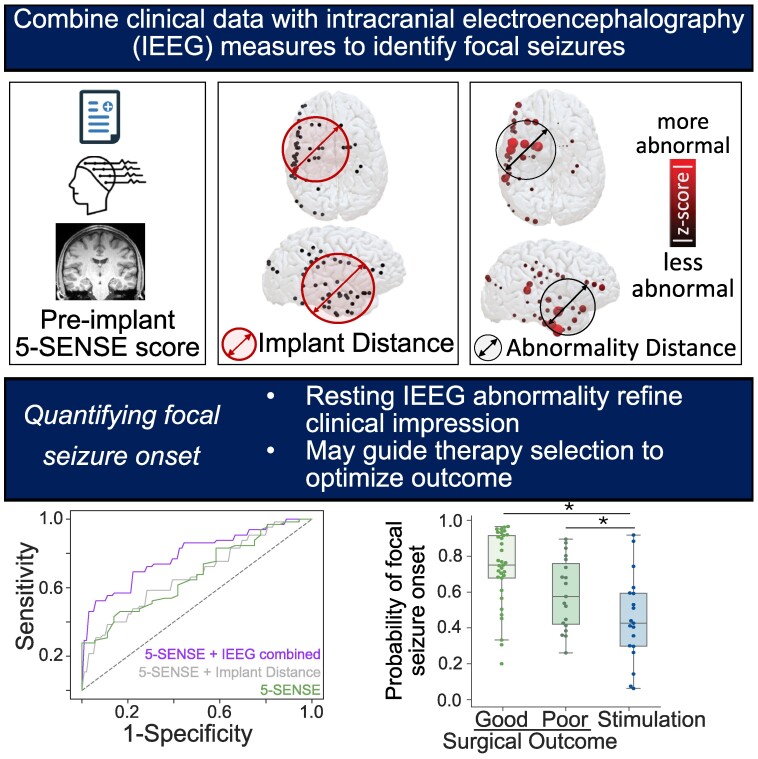

See Thomas, Jaber and Frauscher (https://doi.org/10.1093/braincomms/fcae349) for a scientific commentary on this article.

## Introduction

Over 20 million people worldwide have drug-resistant epilepsy, in which seizures are resistant to medical therapy.^1,2^ Surgery can eliminate or markedly reduce seizures in these patients, but unfortunately, 40–60% of patients who undergo surgery do not become seizure free.^3-6^ Modest outcomes from epilepsy surgery and variability in treatment across centres are driving researchers to develop rigorous quantitative methods and biomarkers to guide invasive treatment.^7-9^ Prior work has focused on several major questions: *Is there a single focal seizure-generating region, or is one present even if we have not located it? What is the location of the generator(s)? What are the dynamics, rate and pattern of seizure spread? And where and how should we intervene?*^7,8,10-15^ In this study, we concentrate on the basic question of whether there is a single focal seizure generator, which is essential to decide whether therapy should be focal, such as surgical resection or ablation, or less targeted, in the form of an implantable device.

Standard practice is to answer the above questions at a multidisciplinary surgical conference, where IEEG, brain imaging and supportive metadata are reviewed manually. Unfortunately, the outcomes of these discussions vary from centre to centre, making it difficult to aggregate data across institutions for retrospective analysis and prospective clinical trials. A number of groups, including ours, are working on quantitative methods to guide therapy, but to our knowledge, none have yet performed strongly enough or been validated in prospective, large-scale trials to be used in routine clinical practice.^9,16^ An important publication by Astner-Rohracher *et al*.^17^ recently suggested the need to compare new methods for guiding therapy against clinical predictions based on the pre-implant data alone. The group’s ‘5 Sense Score’ was able to predict seizure focality in patients prior to invasive electrode implantation with an area under the receiver operating characteristic curve (AUC) of 0.67. This modest but important result validates the need to proceed with IEEG recording in these patients, is easy to apply across centres and provides a floor of accuracy (null model) against which additional methods should be benchmarked to demonstrate value.

In emphasizing quantitative measures, our group and others hope to give clinicians rigorous, reliable tools to aid in clinical decision-making, to improve our understanding of human epileptogenic networks and to improve standardization across centres that will facilitate large-scale, multi-centre clinical trials of existing and new invasive therapies as they arise.^8,9,16^ As quantitative methods are typically compared to clinical experts, we chose to compare our results to expert determinations of seizure focality, choice of therapy and patient outcome.

Clinicians localize epileptic networks with IEEG by inducing seizures to measure their onset and spread patterns (ictal recording). Although interictal or seizure-free IEEG data are not routinely used to classify epilepsy during surgical evaluation, quantitative analysis of interictal segments has proven valuable for localization.^18-22^ Additionally, exclusively using ictal data for localization in the acute inpatient setting may be misleading.^23,24^ While there have been several attempts to use quantitative interictal IEEG biomarkers to predict outcomes following epilepsy surgery, using these measures to look at the question of whether these networks are focal or distributed remains unexplored. Addressing this question may aid clinicians’ decisions between focal intervention and generalized neuromodulation and reduce iatrogenic seizure burden and length of stays in epilepsy monitoring units (EMUs).

## Materials and methods

In this study, we aimed to determine whether quantitative analysis of interictal IEEG data could improve the pre-implant estimate to predict the focality of epileptic networks on IEEG. We hypothesized that the spatial dispersion of abnormalities, quantified through interictal IEEG using a normative atlas, would correlate with the focality of epileptic networks.^18,25^ To test this hypothesis, we classified patients into either focal or non-focal groups based on clinical assessments of IEEG focality. The focal group comprised patients with a focal seizure onset zone on IEEG, while the non-focal group included those with bilateral, multi-focal, broad or non-localized seizure onset zones. We first implemented the 5 Sense Score, which summarizes non-invasive clinical variables to predict focality.^17^ Then, we incorporated a measure quantifying the spatial density of both the implanted IEEG electrodes as well as their associated abnormalities. We used the 5-SENSE score with our spatial IEEG measures in a model to generate cross-validated predictions of focality and evaluated whether that combined model was better than the 5-SENSE score alone. Finally, we evaluated whether the predictions differ according to the ultimate therapy and surgical outcome, suggesting that poor outcomes and palliative therapies are more common in patients with non-focal epileptic networks.

### Patient information

We analysed 101 drug-resistant epilepsy patients from The Hospital of the University of Pennsylvania who underwent pre-surgical evaluation and IEEG with clinical, imaging and electrophysiologic data collected and available for retrospective review. Patients were enrolled serially between 2011 and 2021 after providing written informed consent for IEEG data analysis, in line with the University of Pennsylvania’s IRB-approved protocol (reference number 821778). Patient characteristics are shown in Table 1.

**Table 1:** Patient characteristics

	Non-focal (*n* = 36)	Focal (*n* = 65)	*P* ^a^
Female, *n*	22	30	0.17
Male, *n*	13	35	
Age of onset, mean (std)	17.6 (12.5)	16 (12.4)	0.53
Age at implant, mean (std)	36.6 (10.6)	35.5 (12.2)	0.50
MRI lesional, *n*	17	42	0.13
Focal lesion	5	28	0.02
Non-focal lesion^b^	12	14	
Gray matter channels, mean (std)	45.3 (14.8)	37.82 (17)	0.06
Grids/strips/depths, *n*	8	26	0.13
SEEG	27	39	
Surgery, *n*	11	52	<0.001^c^
Ablation	6	24	0.82
Resection	5	29	
Surgical outcomes (evaluated at 2 years)			
ILAE 1–2	5	28	1.0
ILAE 3–6	3	16	
Neurostimulation device, *n*	15	7	

^a^ *χ* ^2^ for categorical data and U-test for continuous.

^b^Non-focal MRI lesion includes multi-focal, broad and bilateral lesions.^17^

^c^Comparison of surgery versus device.

### Clinical data extraction

#### Non-invasive pre-surgical evaluation phase

We curated information from each patient’s written clinical notes on the following: (i) scalp EEG seizure laterality and localization; (ii) scalp EEG spike laterality and localization; (iii) MRI lesion status, laterality, localization and type; (iv) neurophysiological testing for language dysfunction lateralization; and (v) semiology lateralization and localization. These non-invasive clinical variables aided in formulating hypotheses about the localization and lateralization of epileptic networks.^17^ We then summarized these variables using the recently proposed 5 Sense Score,^17^ which combines five pre-implant clinical variables that were found to predict stereo-EEG (SEEG) focality: (i) extent of the lesion on MRI, (ii) extent of the ictal discharge on scalp EEG, (iii) extent of interictal epileptiform discharges (IEDs) on scalp EEG, (iv) strength of localizing semiology and (v) localization of neuropsychological deficit. More details on the 5-SENSE score are available elsewhere.^17^

#### Invasive IEEG evaluation phase

Following the previous non-invasive phase, IEEG electrodes were implanted to target the epileptic network. Patients were admitted to EMU for IEEG implantation, and seizures were induced to determine the laterality and localization of seizure focus. From discharge summaries and multidisciplinary conference notes, we determined whether the IEEG-defined seizure onset zone was classified as focal, bilateral, multi-focal, broad, non-localized or missed. Based on these data, 65 patients were focal, and 36 were non-focal. The non-focal group included those with bilateral, multi-focal (two or more discrete onset zones), broad (lobar or multi-lobar), non-localized or missed seizure onset zones.

#### Post-implant therapy selection, surgery and follow-up phases

The choice of therapy—resection, ablation or stimulation—was determined by weighing the risk–benefit ratio between achieving seizure freedom and potential neurological or cognitive deficits. Generally, patients found to have focal seizure onset on IEEG received surgery, and patients categorized as non-focal received neuromodulatory devices, but exceptions were seen: patients with suspicion for multi-focal seizures underwent surgery if a key node was thought to be mediating, and focal patients underwent device implantation if seizure onset occurred in the eloquent cortex. Post-surgical ILAE outcomes were derived at 2 years. Table 1 summarizes the surgical outcome and therapy data.

### IEEG electrode reconstruction on MRI

We implemented iEEG-recon, a modular software tool, to reconstruct IEEG electrode contacts on pre-implant T_1_-weighted MRI images.^26^ This tool enables automatic labelling, registration and reconstruction of iEEG electrode coordinates on brain images. In brief, each electrode contact was semi-automatically labelled on CT images, which were then registered to pre-implant T_1_-weighted MRI. Brain segmentation and parcellation were conducted in each subject’s native space using FreeSurfer.^26^ Electrodes were classified as being in grey matter, white matter or outside the brain. We excluded all electrodes located in white matter and outside the brain. For all grey matter electrodes, we determined their corresponding regions based on the Desikan–Killiany parcellation scheme in the patient’s native space.

### IEEG data processing

We selected 20 one-minute interictal IEEG clips for each patient’s stay in the EMU.^27^ These clips were randomly chosen from awake periods throughout the entire recorded EMU stay, excluding 3 days post-implantation and 2 h from any seizure annotation to minimize implant effects and peri-ictal changes during awake periods. To detect awake periods in iEEG, we applied an alpha/delta power ratio (normalized threshold = −0.40) detector, previously described for identifying the highest probability of wakefulness.^28^ We obtained these clips from IEEG.org and applied bipolar referencing. The data were processed using a third-order Butterworth bandpass filter between 0.5 and 80 Hz, a 60 Hz notch filter, and down sampled to 200 Hz. We calculated relative band power (IEEG spectral activity) and magnitude squared coherence (IEEG connectivity) in 5 canonical bands (delta 0.5–4 Hz, theta 4–8 Hz, alpha 8–12 Hz, beta 12–30 Hz and gamma 30–80 Hz), as well as total broadband (0.5–80 Hz) power and coherence, using a 2 s Hamming window with a 1 s overlap. These preprocessing steps are identical to those in our previous work,^18^ with the only exception being that we selected 20 interictal IEEG clips and performed the analysis on all 20 clips to ensure temporal robustness of our quantitative methods.

### Quantifying interictal IEEG abnormalities from normative IEEG atlas

Normative modelling is an innovative case–control approach that quantifies abnormalities in a patient’s IEEG signals by determining how deviant they are from the normal range expected in controls. As IEEG is rarely implanted in patients without epilepsy, we created a normative IEEG atlas by concatenating 2304 electrodes implanted outside epileptogenic tissues, which were presumed healthy, across 144 patients. This normative IEEG atlas has been described in our previous work and is publicly available.^18^ By continuing to research the utility of normative IEEG modelling, we hope to encourage further contribution to a cross-centre data set that represents a greater distribution of ‘normal’ and ‘abnormal’ epileptic IEEG recording.

We quantified interictal IEEG abnormalities from both IEEG spectral activity and connectivity. Briefly, for each IEEG contact’s relative band power in each canonical frequency band, we calculated the *Z*-score from the electrodes in the same parcellated region of interest (ROI) in the normative IEEG atlas. Similarly, for iEEG coherence connectivity between two ROIs in each frequency band, we computed the *Z*-score of equivalent connections between the same ROIs in the normative iEEG atlas. *Z*-score represents the deviation from normal IEEG spectral activity and coherence. We repeated these steps for all 20 one-minute interictal clips, computing a distribution of *Z*-scores for iEEG activity and connectivity. These steps are identical to those in our previous work demonstrating the relationship of these calculated abnormalities to the epileptogenic zone and are further explained in detail there.^18^

### Quantifying spatial coverage of IEEG and spatial dispersion of IEEG abnormalities

The pre-implant clinical hypothesis regarding the focality of the epileptic network influences the spatial coverage of the IEEG implant. In cases where epileptic networks are likely focal, the density of IEEG may be more concentrated in areas believed to be involved in seizure generation and propagation. Conversely, if clinicians suspect a non-focal epileptic network, the IEEG coverage is likely to be broader.

To quantify this clinical intuition about the focality of the epileptic network, we computed standard distance (SD) between IEEG contacts. SD represents the 3D standard deviation of each patient’s IEEG electrode coverage.^29^ Mathematically, if an IEEG contact *i* has coordinates (*x*, *y*, *z*) and there are *n* contacts in a patient, the SD can be computed as follows:


$$\mathrm{SD}\;=\;\sqrt{\frac{\sum_{i=1}^{n}\;{\left(x_{i}-\bar{X}\right)}^{2}}{n}+\frac{\sum_{i=1}^{n}\;{\left(y_{i}-\bar{Y}\right)}^{2}}{n}+\frac{\sum_{i=1}^{n}\;{\left(z_{i}-\bar{Z}\right)}^{2}}{n}}$$

A high SD value indicates a wider coverage, while a smaller SD value represents a more compact coverage. We refer to the SD between IEEG contacts as the ‘implant distance’.

To compute the spatial dispersion of IEEG abnormalities, we implemented weighted SD (WSD). WSD weights the SD with the abnormality values of each iEEG contact. Mathematically, if the abnormality of an IEEG contact *i* with coordinates (*x*, *y*, *z*) is *w*, and there are *n* contacts in a patient, the WSD can be computed as follows:


$$\mathrm{WSD}\;=\;\sqrt{\frac{\sum_{i=1}^{n}\;w_{i}{\left(x_{i}-{\bar{X}}_{w}\right)}^{2}}{\sum_{i=1}^{n}\;w_{i}}+\frac{\sum_{i=1}^{n}\;w_{i}{\left(y_{i}-{\bar{Y}}_{w}\right)}^{2}}{\sum_{i=1}^{n}\;w_{i}}+\frac{\sum_{i=1}^{n}\;w_{i}{\left(z_{i}-{\bar{Z}}_{w}\right)}^{2}}{\sum_{i=1}^{n}\;w_{i}}}$$

where {${\bar{X}}_{w}$, ${\bar{Y}}_{w}$, ${\bar{Z}}_{w}$} represent the weighted mean centre:


$${\bar{X}}_{w}=\;\frac{\sum_{i=1}^{n}\;w_{i}x_{i}}{\;\sum_{i=1}^{n}\;w_{i}}$$

**Figure 1:**
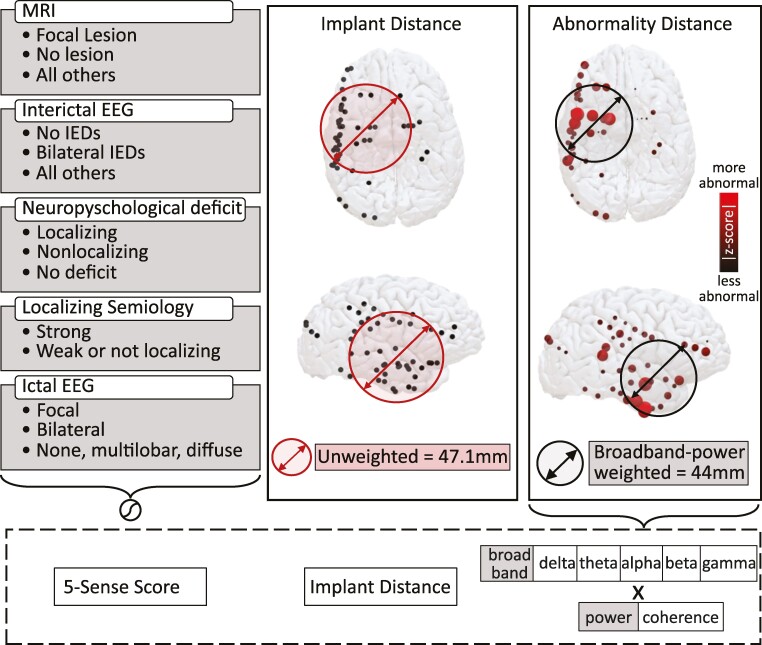
**Features calculated to discriminate patients found to have focal from non-focal seizure onset with IEEG.** Left: 5-SENSE score using pre-implantation data previously demonstrated to have predictive value. IED, interictal epileptiform discharge. Middle: SD (*implant distance*), calculated as the standard deviation of distance from each electrode to the mean centre of the electrodes in space. Right: WSD (*abnormality distance*), calculated as the standard deviation of the dispersion of iEEG abnormality for each of six bands in both band power and coherence. We refer to these interictal iEEG features as the ‘sixth sense’.

In simpler terms, the WSD takes into account the abnormality value at each IEEG contact and measures the spatial distribution of abnormality. A high WSD value indicates that abnormalities on IEEG are spatially widespread, while a low WSD value suggests that the abnormalities are located spatially closer together. We refer to the WSD of IEEG abnormalities as the ‘abnormality distance’. The concepts described above are illustrated schematically in the overview presented in Fig. 1.

### Statistics

We conducted univariate analysis to test how each metric discriminated focal from non-focal iEEG-seizure onset. In these univariate tests, we calculated effect sizes using Cohen’s *d* score, non-parametric effect sizes using the AUC with 95% confidence intervals (CIs) and non-parametric two-tailed *P*-values from the Mann–Whitney U-test. We combined these quantitative metrics using multivariate logistic regression with lasso regularization, to mitigate the impact of correlated features and reduce the number of features. We applied leave-one-out cross-validation to predict IEEG focality in unseen (test) patients. We measured the AUC of these predictive models to score and compare overall performance, acknowledging that AUC does not equate to sensitivity or specificity, and better performance in one measure does not imply better performance in the others. We applied DeLong’s test to the difference in AUCs to compare the different models for our primary outcome, aiming to test our hypotheses whether a model combining IEEG abnormality distances, implant distance and the 5-SENSE score improved upon both only the 5-SENSE and combining the 5-SENSE with implant distance. We applied Holm–Bonferroni correction at a significance level of 5% to correct for these sequential multiple comparisons. We report the sensitivity and specificity from the binary class predictions of our logistic regression model, compared to the bootstrapping method weighted for specificity in developing the 5-SENSE score cut-off. We performed all analyses with Python 3.10 and default regularization parameters for logistic regression (‘l1’ norm, *C* = 1) in sklearn.

## Results

One hundred and one patients met the inclusion criteria. Clinicians determined that seizure generators were focal in 65 patients and non-focal in 36 patients. Among the 36 non-focal patients, 11 underwent surgery and 15 received stimulation therapy. Out of the 65 focal patients, 52 had surgery and 7 were treated with stimulation therapy. Table 1 provides an overview of surgical outcomes and therapy information. Our primary research questions were as follows: (i) Can quantifying abnormalities in interictal IEEG differentiate between focal and non-focal epileptic networks? And (ii) how do interictal quantitative predictions differ according to the choice of therapy and patient outcome?

### Quantitative features distinguish focal and non-focal epileptic networks

**Figure 2:**
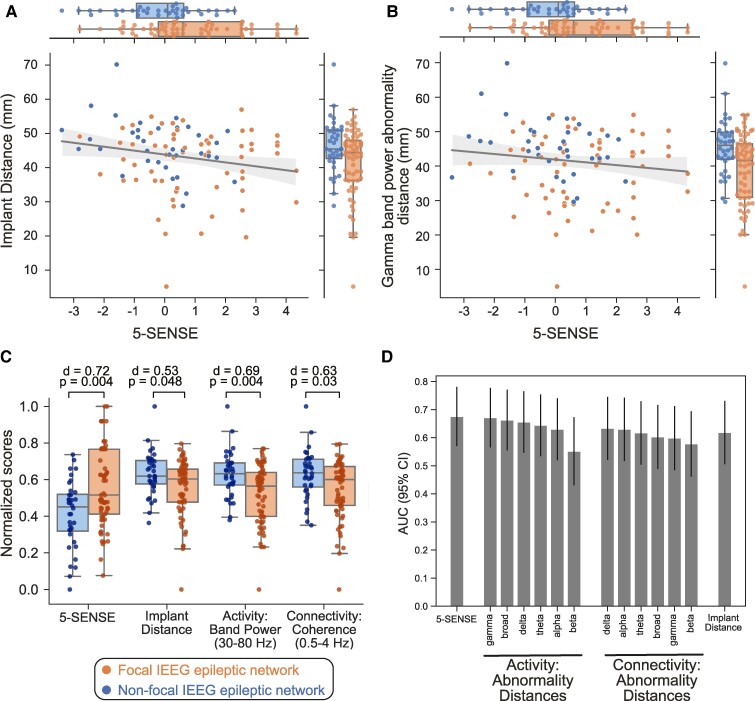
**Determining focality of epileptic networks from quantitative pre-implant and IEEG features.** Each dot in the scatter plots represents a patient. (**A**) Implant distance and 5-SENSE score discriminate patients with focal and non-focal epileptic networks, but they have a weak negative correlation (*r* = −0.22, *P* = 0.024) with each other. (**B**) Spatial dispersion of IEEG spectral abnormalities in the gamma band can differentiate focal and non-focal epileptic networks; these IEEG abnormality features are not correlated with 5-SENSE score (*r* = −0.16, *P* = 0.11). (**C**) Individually, the 5-SENSE score, implant distance and spatial dispersion of IEEG abnormalities distinguish focal and non-focal epileptic networks. Each point represents the normalized value for one patient by each variable. Cohen’s *d* and *P*-values of Mann–Whitney U-test compare focal and non-focal patients for each measure. (**D**) Bar plot showing the discrimination power in AUC ± 95% CI of each quantitative feature in differentiating focal and non-focal epileptic networks.

We conducted univariate analyses on (i) 5-SENSE score, (ii) spatial coverage of IEEG (implant distance) and (iii) spatial dispersion of abnormalities quantified from interictal IEEG activity and connectivity features (abnormality distance). We investigated the association of these variables to determine IEEG focality. As depicted in Fig. 2, these variables significantly distinguish patients with focal epileptic networks from those with non-focal networks. Among these variables, as shown in Fig. 2C and D, the 5-SENSE score had the highest effect size [*P* = 0.004, *d* = 0.72, AUC = 0.68 (95% CI 0.57, 0.78)], followed by the abnormality distance in gamma band power [*P* = 0.004, *d* = 0.69, AUC = 0.68 (95% CI 0.57, 0.78)].

Although these variables could independently distinguish between focal and non-focal patients, they exhibited a weak correlation to the 5-SENSE, as shown in Fig. 2A and B. In particular, the correlation between the 5-SENSE score and the implant distance was *r* = −0.22 (*P* = 0.024), implying that patients with a higher pre-implant probability of focality had a less spatially dispersed implant. The correlation between the 5-SENSE score and the abnormality distance in the gamma band was *r* = −0.13 (*P* = 0.19), implying a non-significant trend towards lower gamma band abnormality dispersion in patients with a higher pre-implant probability of focality. The combination of low correlation among variables and their individual discriminatory ability in determining focal and non-focal epileptic network suggests that these variables may have complementary information when combined.

### Predicting focality by combining pre-implant and interictal IEEG data

To predict IEEG focality, we conducted cross-validated logistic regression classification using three distinct models: (i) model incorporating only the 5-SENSE scores, (ii) model combining features from 5-SENSE scores and implant distance and (iii) model combining features from 5-SENSE scores and IEEG abnormality distances.

**Figure 3:**
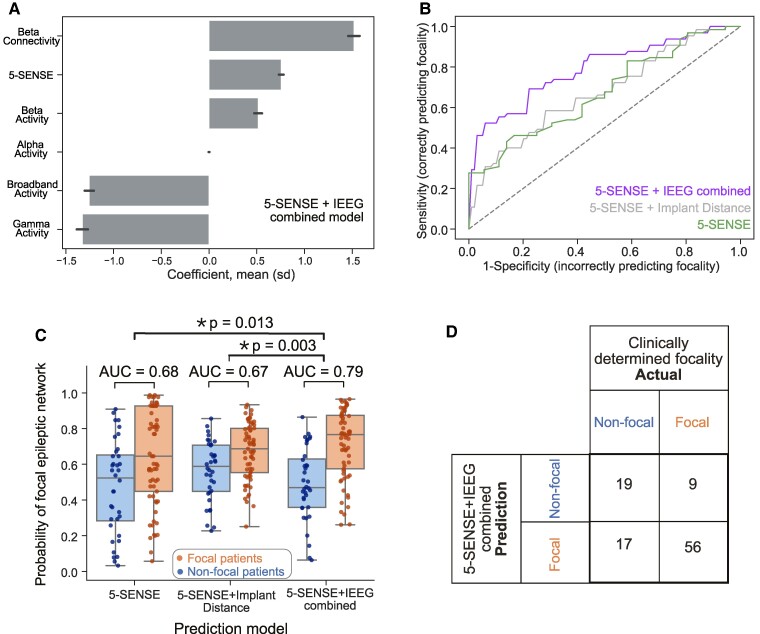
**Multivariable model combining IEEG features with the 5-SENSE score to predict IEEG focality.** (**A**) Features combine in a linear model resulting in AUC = 0.79 to predict focality. Mean ± SD of feature coefficients across cross-validation folds with lasso regularization. (**B**) Comparison of ROC curves of the predictions of the 5-SENSE + IEEG model to a pre-implant baseline model incorporating only 5-SENSE + implant distance and the 5-SENSE score. (**C**) Each point displays the prediction from a single patient for each model. 5-SENSE scores combined with interictal IEEG abnormality distances predict IEEG focality most accurately [cross-validated AUC 0.79 (95% CI 0.70, 0.88)], outperforming 5-SENSE score alone [AUC 0.68 (95% CI 0.57, 0.78), DeLong’s test *P* = 0.013, Holm corrected = 0.027] and its combination with implant distances [AUC 0.67 (95% CI 0.57, 0.78), DeLong’s *P* = 0.003, Holm corrected = 0.009]. (**D**) Confusion matrix of the binary predictions from the model incorporating IEEG features and the 5-SENSE score.

Figure 3 shows the comparison of these three models, along with the prediction performance for each. We found that the combination of 5-SENSE scores and interictal IEEG abnormality distances predicts IEEG focality most accurately, with a cross-validated AUC of 0.79 (0.70, 0.88) (Fig. 3B and C). This outperformed the AUC of the 5-SENSE score alone by DeLong’s test (Holm-corrected *P* = 0.027). The 5-SENSE score, in combination with only the implant distances, achieved a cross-validated AUC of 0.68 (0.57, 0.78), which did not significantly outperform the 5-SENSE alone (Holm-corrected DeLong’s *P* = 0.96). The model combining 5-SENSE, implant distance and abnormality distances outperformed the model combining only 5-SENSE with implant distances (DeLong’s *P* = 0.003, Holm corrected = 0.009). This indicates that the added information of iEEG abnormalities may be driving the improvement in the combined model and not just the implant distance. IEEG abnormality distances were among the most important features of the combined model (Fig. 3A). The combined model predicted focality with sensitivity of 86% and specificity of 53% at the optimal operating point of the receiver operator characteristic (ROC) curve (Fig. 3D).

### Focality prediction relates to surgical outcomes and implantation types

We next examined whether the outputs of our model, trained to predict focality from interictal iEEG data, varied in subgroup analysis by surgical outcome, implantation type or subtypes of non-focal epilepsy.

**Figure 4:**
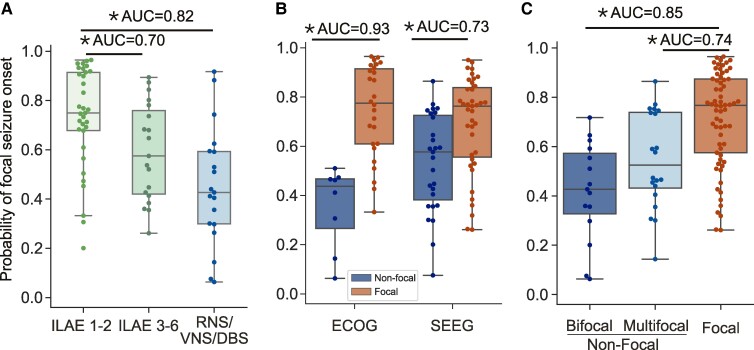
**Predictive value of the 5-SENSE model combined with IEEG for focality and its implications for surgical outcomes and implantation types.** Each point displays the prediction from a single patient for the combined model by specified subgroup. (**A**) Model indicates a higher likelihood of focal epileptic networks in patients achieving post-surgery seizure freedom compared to those who continued to experience seizures after surgery [AUC = 0.70 (95% CI 0.56, 0.85), U-test *P* = 0.015, Holm-corrected *P* = 0.03] and to those who underwent neurostimulation [AUC = 0.82 (95% CI 0.70, 0.94), U-test *P* < 0.001, Holm-corrected *P* < 0.001]. (**B**) Greater effect size in focal versus non-focal networks for patients with ECOG implants [AUC = 0.93 (0.85, 1.00) U-test *P* < 0.001] than SEEG implants [AUC = 0.73 (0.61, 0.85), U-test *P* = 0.002]. (**C**) Among ‘non-focal’ patients, focality prediction significantly differs between bifocal and unifocal [AUC = 0.85 (0.77, 0.94), U-test *P* < 0.001, Holm-corrected *P* < 0.001] and multi-focal and unifocal epileptic networks [AUC = 0.74 (0.63, 0.85), *P* = 0.001, Holm-corrected *P* = 0.003] with no significant difference between bifocal and multi-focal patients. Asterisk indicates *P* < 0.05 of Mann–Whitney U-test. RNS, responsive neurostimulation; VNS, vagus nerve stimulation; DBS, deep brain stimulation.

Figure 4A shows that the predictions of focality combining 5-SENSE score and IEEG were higher in patients who achieved seizure freedom post-surgery than those who continued to experience seizures after surgery [AUC = 0.70 (95% CI 0.56, 0.85), U-test *P* = 0.015, Holm-corrected *P* = 0.03] and those who underwent neurostimulation [AUC = 0.82 (95% CI 0.70, 0.94), U-test *P* < 0.001, Holm-corrected *P* < 0.001]. This suggests that more focal networks are associated with better surgical outcomes. Figure 4B shows that the effect size between focal and non-focal epileptic networks was greater in patients with electrocorticography (ECOG) implants [AUC = 0.93 (95% CI 0.85, 1.00)] compared to those with SEEG implants [AUC = 0.73 (95% CI 0.61, 0.85)]. When looking at ‘non-focal’ patients by subcategories, Fig. 4C shows that quantitative predictions of focality significantly differ between patients with bifocal seizure onsets and those with unifocal seizure onset [AUC = 0.85 (95% CI 0.77, 0.94), U-test *P* < 0.001, Holm-corrected *P* < 0.001] and patients with multi-focal onsets and those with unifocal onset [AUC = 0.74 (95% CI 0.63, 0.85), *P* = 0.001, Holm-corrected *P* = 0.003].

Supplementary Fig. 1 shows consistent results as in Fig. 4A for seizure outcomes measured on the Engel scale and for different predictive models, though the ability to predict seizure freedom may be stronger when the model includes IEEG abnormalities. Supplementary Fig. 2 shows consistent results as in Fig. 4B and C for different choices of predictive models. Collectively, these findings provide additional validation of our approach, indicating that the predicted probability of a focal epileptic network tends to be lower in cases where therapies are palliative, or when the IEEG has a broader spatial coverage.

## Discussion

In this study, we present a quantitative method to categorize the focality of epileptic networks for individual patients based upon their IEEG, by comparing their data to an atlas of prior patients. The method combines (i) pre-implant clinical variables, (ii) spatial coverage of IEEG implant and (iii) spatial dispersion of abnormalities quantified on interictal IEEG segments. Our findings suggest that we can combine these variables to predict whether a patient has a focal or a distributed epileptic network. These findings have significant clinical implications. Patients with a high probability of having a focal epileptic network might be better candidates for focal surgical intervention, such as laser ablation or surgical resection, and require more complex analyses, such as from ictal recordings. Conversely, patients with distributed or multi-focal epileptic networks, and currently a lower likelihood of seizure-free outcome, might benefit more from palliative neurostimulation therapies that reduce seizure frequency and/or severity, but with a much lower chance of rendering them seizure free. In these cases, our method might suggest that clinicians consider regional or central neurostimulation therapy earlier, rather than ‘chasing’ poorly localized ictal onset patterns. Since our method does not rely on ictal recording, it has the potential to reduce length of stay during implantation, reduce morbidity from seizures associated with medication withdrawal and reduce cost in patients found unlikely to have a clear focal onset.

Of interest, our computation of the 5-SENSE score replicated the findings of the original study in AUC. This reinforces that study’s generalizability to other tertiary care centres. In an interesting confirmatory result, we found that a quantitative measure of the spatial dispersion of implanted IEEG electrodes predicted network focality with similar precision as the 5-SENSE score, indicating that in our centre, the implant strategy accurately translated the pre-implant data into the next phase of pre-surgical evaluation. We found that a model incorporating pre-implant data with the spatial dispersion of IEEG interictal abnormality improved significantly upon estimates of whether a given patient’s network demonstrates a focal or non-focal seizure onset pattern.

Perhaps unsurprisingly, given the relationship between the clinical definition of focality and the decision to recommend focal surgery versus neuromodulation via an implantable device (Table 1), we found that predictions of focality were effective in differentiating whether patients were referred to resective surgery or ablation, or for device therapy (Fig. 4). Generally, ‘focal’ patients received surgery and ‘non-focal’ patients received neuromodulatory devices in our cohort though exceptions exist clinically; for example, patients with focal seizure onset in eloquent cortex or in regions anatomically excluded from resection (e.g. because of vascularity or access issues) may receive neuromodulation.^30^ In addition, patients with less localized seizures may have received surgery if key nodes were thought to be most implicated, as inferred from seizure spread or for other reasons.^12,13^ While our model does not include these more advanced considerations, it does provide a framework for including other quantitative measures, and it also provides a benchmark to assess whether these more subjective decisions improved outcome more than quantitative measures of focality alone.

In addition, we observed that quantitative models of focality could differentiate between favourable and unfavourable surgical outcomes (Fig. 4). All models (5-SENSE, 5-SENSE + Implant distance, combined 5-SENSE + IEEG) discriminated ILAE 1 or 2 from ILAE 3+. However, only the IEEG model discriminated Engel 1 from Engel 2 + outcomes. Presumably, broader epileptogenic networks may be less technically amenable to ablation or resection therapy, but an exact mechanism by which the spatial dispersion of abnormality relates to outcomes in surgical patients also remains unclear, and it is likely a function of the specific physiology of each patient.^10,12,13,31,32^ Interestingly, abnormality distances both in relative power/coherence bands and in absolute broadband power were found to be important predictors in the combined model. Nonlinear interactions across frequency bands may play a role in the focality of seizure onset zones,^33-35^ which would likely require larger data sets to disentangle with more complex models.

The practice of our centre over the time course of this study reflects wider trends in North America towards more SEEG implantation and less ECOG.^36^ We observed that all models performed better in discriminating focal from non-focal networks in patients implanted with subdural ECOG electrodes compared to patients implanted with SEEG, including the 5-SENSE score, which was developed on an entirely SEEG population. This phenomenon may reflect selection bias surrounding which patients were chosen for subdural ECOG implants versus SEEG implants: Subdural ECOG implants are often used to delineate seizure onset regions from eloquent cortex, often around focal lesions, whereas patients undergoing SEEG often receive broad cortical and subcortical sampling, potentially reflecting a more diffuse pre-implant hypothesis.

The need to localize seizure onset and spread, beyond the baseline clinical hypothesis or our analysis of background interictal abnormality, warrants further study and rigorous quantification to incorporate these measures other biomarkers of epileptogenicity with our interictal and implant spatial measures. The literature here is more limited, relating onset region and time of spread to other regions, and its impact on eventual outcome are best documented for medial temporal lobe epilepsy. For example, a study by Andrew *et al*.^11^ demonstrates that seizures that spread rapidly outside the resection margin associated with a poorer outcome after temporal lobectomy. The literature is less clear for seizures that spread around focal lesions and to other structures with strong connectivity, for example seizures that spread rapidly from mesial temporal lobe to insula. The additional degrees of freedom in these types of analyses will require a much larger number of patients in an atlas of ictal recordings, interventions and known outcomes in order to quantify and understand this process.

An advantage of the interictal method presented in the current study is potentially limiting the amount of required clinical data in EMU stays. The method requires only 20 min of interictal IEEG data to map focality. Should a non-focal network be predicted, this could obviate the need for prolonged ictal recording and facilitate more expeditious neuromodulation treatment. Increasing literature measuring interictal brain abnormalities^18,19,32,37^ and perturbing networks with stimulation^38-40^ may lead invasive intracranial studies to rely less on eliciting spontaneous seizures in the future to identify the major areas of dysfunction in epileptic networks amenable to treatment. It is likely that some combination of these approaches, with quantitative metrics of seizure dynamics, incorporating data on brain regions of interest and spatial distance, will likely be needed to improve on the present model. Central to this improvement is the need to aggregate and collaboratively analyse data from many patients in many centres using standardized protocols. We hope that using large numbers of patients can compensate for sparse electrode sampling in each patient.

Ultimately, predictive computational models of epileptogenicity may help guide surgical decision-making. In this study, we related the IEEG activity and connectivity of each patient to a corpus of prior patients. We show spatial coverage of electrodes, their associated IEEG abnormalities and the data used in determining that coverage relates to seizure onset extent and surgical outcomes. By this framework, future studies may elucidate the cost–benefit decision analysis of implanting each additional electrode to identify seizure generators, or perhaps obviate the need to move forward with ictal recording in cases where more diffuse network topology is likely.

This study has important limitations. As a single-centre, retrospective cohort study at a tertiary care institution, generalizability to other centres has not yet been determined. We used cross-validated lasso logistic regression to assess out-of-sample performance. There is considerable heterogeneity across epilepsy patients, and while a cohort of 101 patients is relatively large for such studies of IEEG, studies across multiple centres with greater numbers of patients are likely necessary to capture the true variability in ‘non-focal’ onset and give a better sense of generalizability. The 5-SENSE score, in its multi-centre implementation, favoured specificity over sensitivity of the final model. In this analysis, we did not specify this weighting. We found higher sensitivity than specificity in the binary predictions of our model combining preclinical data with spatial abnormality data. This may have clinical relevance in that the 5-SENSE may prevent unnecessary invasive testing with high specificity, whereas including IEEG data may identify the most cases potentially amenable for focal intervention. Notably, the 5-SENSE score has been validated based on a multi-centre study, whereas our model is based on a monocentric approach. Validating the clinical utility of our model will require further testing across multiple centres and tuning for optimal clinical utility depending on the goals of the patient and treatment team.

The use of normative IEEG by identifying uninvolved epileptic electrodes is a nascent field. The openly available atlas we employed likely requires substantially more data to improve the accuracy and generalizability of the models. We assume that unaffected electrodes in patients with epilepsy reflect true ‘normal’ activity and connectivity. This assumption is likely incorrect, as they are derived from epileptic brains, which likely skews the definition of ‘normal’. However, the results of this study and others in normative IEEG modelling show promise in providing measures of epileptogenicity and support the continued use of this approach, for now, and contribution to a central collaborative corpus of IEEG data. It will be important for future studies of interictal IEEG to incorporate other biomarkers of epileptogenicity besides band power and connectivity, such as interictal spikes and high-frequency oscillations.^28,32,41,42^ We acknowledge that collecting the clinical, imaging and electrophysiologic data and extracting the quantitative features needed to perform our analysis are currently too burdensome for most clinicians. Here, we aim to demonstrate the utility in tools that mirror the extensive data analysis currently done qualitatively by clinicians. We ultimately hope to be able to package quantitative approaches such as ours into an easy-to-use automated format.

In conclusion, in this study, we propose rigorous, data-driven quantitative measures to assess the spatial extent of epileptic networks in the brain. The quantitative methods we tested can complement pre-surgical evaluations to decrease lengthy hospital stays and optimize patient and therapy selection in drug-resistant epilepsy. Ultimately, these tools aim to standardize clinical decisions in ways that can be deployed in multi-centre studies to advance and optimize care.

## Supplementary Material

fcae320_Supplementary_Data

## Data Availability

All IEEG data used in this study are publicly available at https://www.ieeg.org/. The normative iEEG atlas can be accessed at https://discover.pennsieve.io/datasets/179. The iEEG-recon software is available at https://ieeg-recon.readthedocs.io. Documented codes and the 5-SENSE score for all patients included in our analysis can be found at https://github.com/penn-cnt/Sixth_Sense.git.
