# Supplementary material for: The sixth sense: how much does interictal intracranial EEG add to determining the focality of epileptic networks?
Source: Brain Commun. 2024 Sep 27;6(5):fcae320. doi: 10.1093/braincomms/fcae320 (PMC11495218; doi:10.1093/braincomms/fcae320)
Supplement: fcae320_Supplementary_Data [file fcae320_supplementary_data.pdf]

Supplementary figure 1

Each data point represents a single patient's focality score by the analyzed subgroup

Top Figure: Focality scores of patients receiving surgery vs device implantation (RNS/VNS/DBS) based on the three scoring systems.

Lower six figures: 2 year outcomes from surgery by ILAE (top) or Engel (bottom) metrics by each of the three analyzed scoring systems, comparing good outcome surgery to poor outcome surgery and to device recipients

Asterisks indicate p-value of Holm-Bonferroni corrected Mann-Whitney U-Test< 0.05

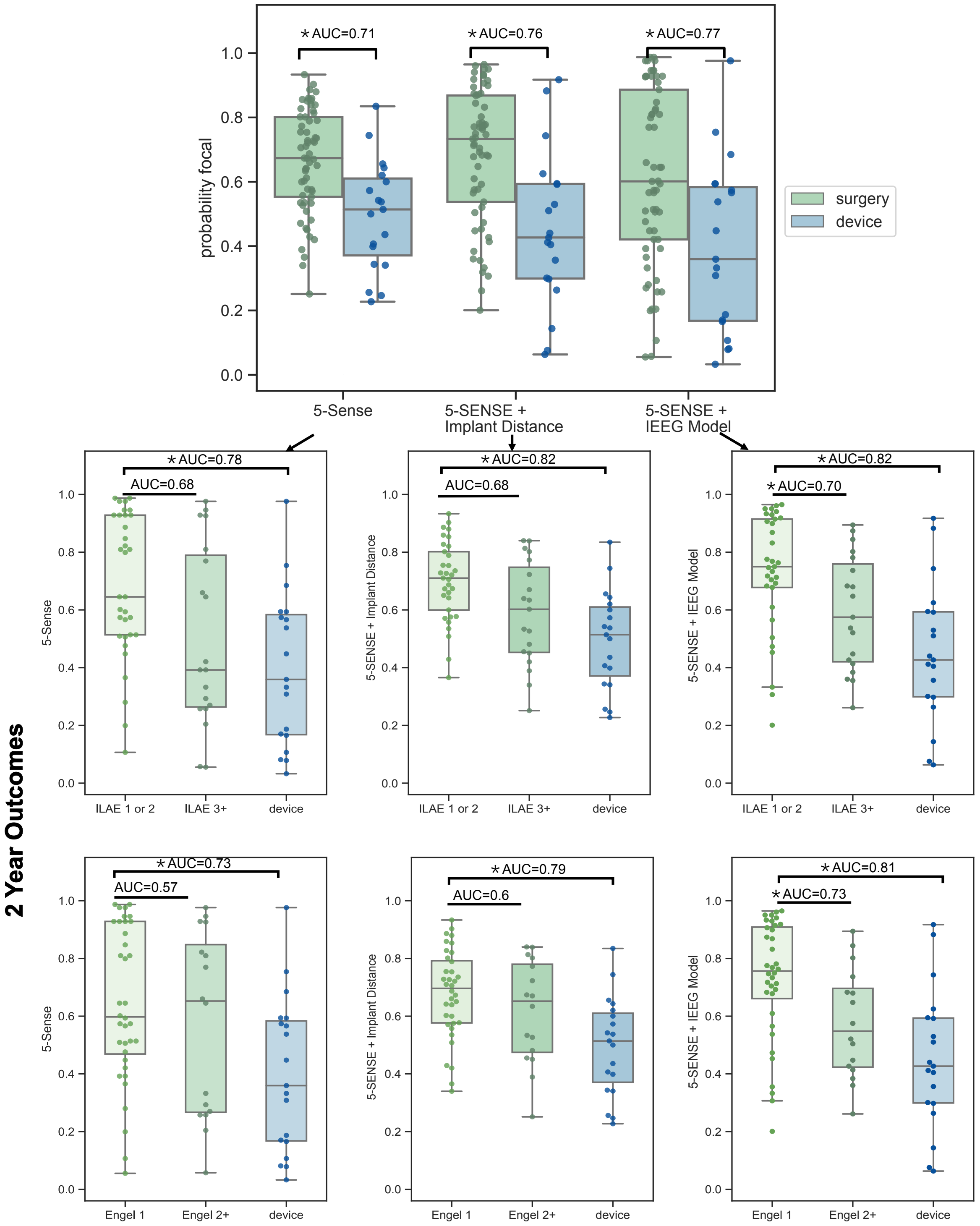

*AUC=0.71
*AUC=0.76
*AUC=0.77
probability focal
surgery
device
5-Sense
5-SENSE + Implant Distance
5-SENSE + IEEG Model
2 Year Outcomes
*AUC=0.78
AUC=0.68
*AUC=0.82
AUC=0.68
*AUC=0.82
*AUC=0.70
5-Sense
5-SENSE + Implant Distance
5-SENSE + IEEG Model
ILAE 1 or 2
ILAE 3+
device
*AUC=0.73
AUC=0.57
*AUC=0.79
AUC=0.6
*AUC=0.81
*AUC=0.73
Engel 1
Engel 2+
device

Supplementary figure 2

Each data point represents a single patient's focality score by the analyzed subgroup.

Subfigures a)-c) display focality scores by grids and strips patients separate from Stereotactic EEG and quality of focality prediction evaluated in each group separately. Panels d)-f) breaks nonfocal into subgroups - bifocal and broad/multifocal – and focality scores in both subgroups are compared to those that were deemed unifocal. p-values displayed are Holm-Bonferroni corrected Mann-Whitney U-tests.

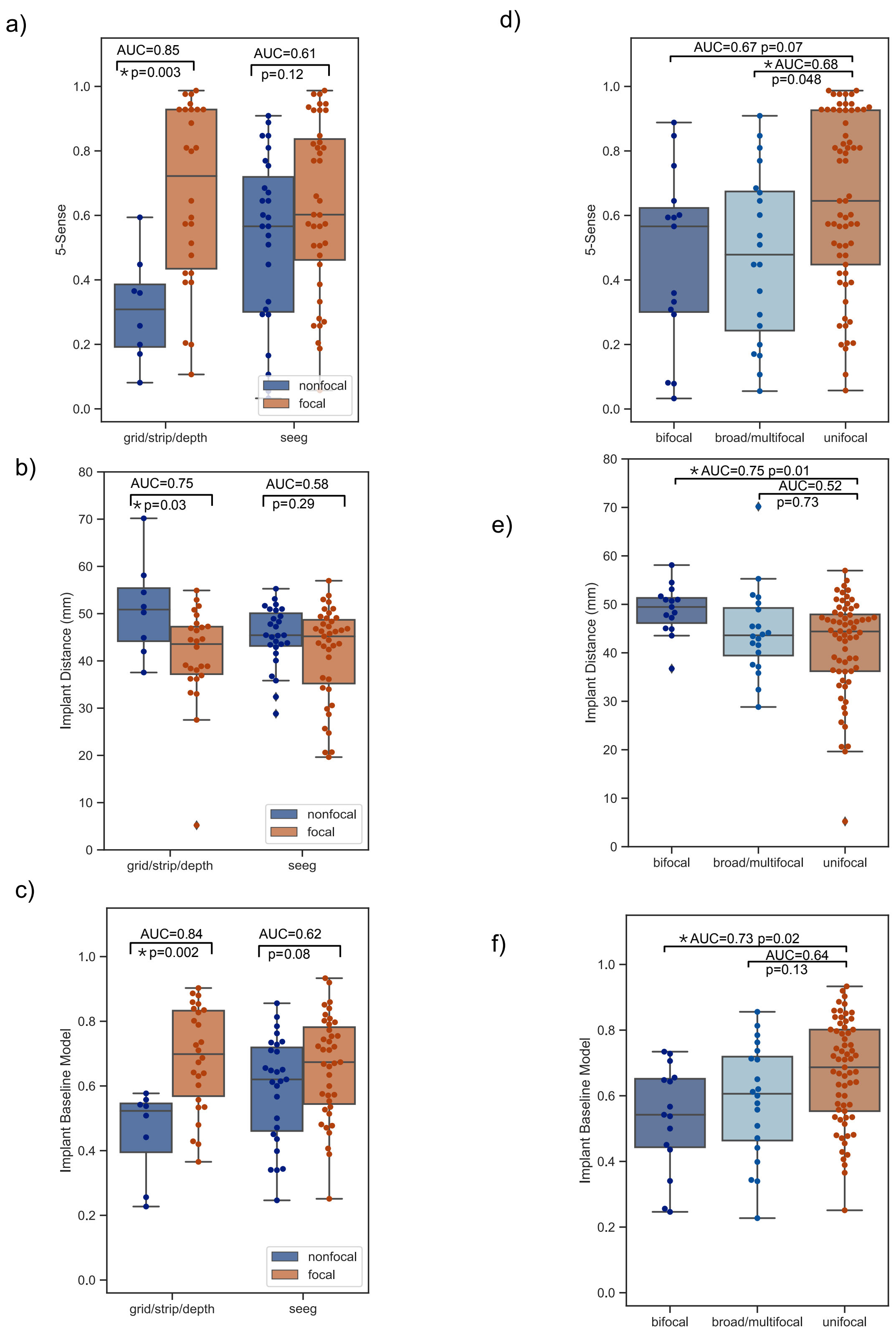

a)
AUC=0.85
*p=0.003
AUC=0.61
p=0.12
5-Sense
nonfocal
focal
grid/strip/depth
seeg
b)
AUC=0.75
*p=0.03
AUC=0.58
p=0.29
Implant Distance (mm)
nonfocal
focal
grid/strip/depth
seeg
c)
AUC=0.84
*p=0.002
AUC=0.62
p=0.08
Implant Baseline Model
nonfocal
focal
grid/strip/depth
seeg
d)
AUC=0.67 p=0.07
*AUC=0.68
p=0.048
5-Sense
bifocal
broad/multifocal
unifocal
e)
*AUC=0.75 p=0.01
AUC=0.52
p=0.73
Implant Distance (mm)
bifocal
broad/multifocal
unifocal
f)
*AUC=0.73 p=0.02
AUC=0.64
p=0.13
Implant Baseline Model
bifocal
broad/multifocal
unifocal
